# Revision rhinoplasty: physician–patient aesthetic and functional evaluation^☆^

**DOI:** 10.1016/j.bjorl.2017.08.011

**Published:** 2017-09-14

**Authors:** Heloisa Nardi Koerner Vian, Cezar Augusto Sarraff Berger, Danielle Candia Barra, Ana Paula Perin

**Affiliations:** a Instituto Paranaense de Otorrinolaringologia (IPO), Curitiba, PR, Brazil; b Associação Paranaense de Otorrinolaringologia (ASPO), Liga de Otorrinolaringologia, Curitiba, PR, Brazil; c Instituto Paranaense de Otorrinolaringologia (IPO), Hospital IPO, Curitiba, PR, Brazil; d Universidade Federal do Paraná (UFPR), Curitiba, PR, Brazil; e Universidade Positivo, Curitiba, PR, Brazil

**Keywords:** Rhinoplasty, Revision rhinoplasty, Rhinoseptoplasty, Rinoplastia, Rinoplastia revisional, Rinosseptoplastia

## Abstract

**Introduction:**

Approximately 5–15% of patients submitted to rhinoplasty operations undergo revision surgery. Those patients have varied functional and aesthetic complaints that should receive a detailed assessment that includes all the expectations the patient had before the previous procedure.

**Objective:**

To draw the profile of the main aesthetic-functional complaints reported by patients to be submitted to revision rhinoplasty and to correlate them with the internal and external objective nasal evaluation performed by the surgeon.

**Methods:**

A prospective study was conducted with 43 patients to be submitted to revision rhinoplasty and their respective surgeons, by applying a questionnaire about the patients’ epidemiological questions and subjective aesthetic-functional complaints as well as the respective functional deformities observed by the surgeons. Subsequently, these data were correlated with the purpose of observing the frequency of congruent reports between physicians and patients.

**Results:**

The presence of drooping tip and residual bridge hump were the patients’ main complaints, confirmed by the surgeons. The correlation between subjective obstructive symptoms and the intranasal evaluation performed by surgeons was shown to be present in 87.5% of the cases. Among the patients with respiratory symptoms, the main deformity identified was residual septal deviation in 56.25% of the cases.

**Conclusion:**

The drooping tip followed by residual hump were the main complaints reported by the patients and confirmed by the objective examination by the physicians. The presence of nasal obstructive complaints in 37.2% of the patients shows that greater attention needs to be paid to functional deformities during the first surgical procedure. The differences observed between patients’ complaints and surgeons’ evaluations confirm the need for detailed assessment and clarification to the patients regarding their expectations and actual surgical possibilities.

## Resumo

**Introdução:**

As rinoplastias possuem índice de revisão em torno de 5% a 15% dos pacientes opera-dos. Tais pacientes possuem queixas funcionais e estéticas variadas e a avaliação detalhada é de extrema importância, tendo em vista todas as expectativas em torno de um procedimento já anteriormente realizado.

**Objetivo:**

Traçar o perfil das principais queixas estético-funcionais referidas pelo paciente a ser submetido à rinoplastia revisional e correlacioná-las a avaliação nasal objetiva interna e externa realizada pelo cirurgião.

**Método:**

Foi realizado um estudo prospectivo com 43 pacientes a serem submetidos à rinoplastia revisional e com seus respectivos cirurgiões, através da aplicação de questionário acerca de questões epidemiológicas e queixas estético-funcionais subjetivas dos pacientes e as respectivas deformidades funcionais observadas pelos cirurgiões. Após, esses dados foram correlacionados com a finalidade de observar a frequência de relatos concomitantes entre os médicos e pacientes.

**Resultados:**

A presença de ponta caída e giba óssea residual foram as principais queixas dos pacientes confirmadas pelos cirurgiões. Já a correlação entre sintomas subjetivos obstrutivos e a avaliação intranasal realizada pelos cirurgiões demonstrou estar presente em 87,5% dos casos. Dentre os pacientes com sintomas respiratórios, a principal deformidade encontrada foi o desvio septal residual em 56,25% dos casos.

**Conclusão:**

A ponta caída seguida de giba óssea residual foram as principais queixas relatadas pelos pacientes e confirmadas ao exame objetivo pelos médicos. A presença de 37,2% dos pacientes com queixas obstrutivas nasais demonstra que maior atenção deve ser dada a deformidades funcionais já durante o primeiro procedimento cirúrgico. As diferenças observadas entre as queixas dos pacientes e avaliações dos cirurgiões comprovam a necessidade da avaliação pormenorizada e esclarecimento ao paciente com relação as suas expectativas e reais possibilidades cirúrgicas.

## Introduction

In otorhinolaryngologic practice, one can observe that a request for revision rhinoplasty by the patients is very frequent, and it is directly related to the number of primary rhinoplasties performed.

The incidence of revision rhinoplasties is far from negligible, with a mean of 5–15% of the primary cases operated, with some articles showing incidences above 21%.^1^, ^2^, ^3^

In most cases these patients have, diverse functional and aesthetic complaints that may necessitate surgery varying from simple procedures such as minor revision, to more extensive corrections that may necessitate the use of varied techniques and grafts.

The thorough evaluation of the patient to be submitted to a revision rhinoplasty is extremely important, since these are patients with much higher expectations compared to when they underwent their primary rhinoplasty, mainly because they are dissatisfied and often even disappointed with the results of the previous surgery.^2^, ^4^

Current studies demonstrate the aesthetic and functional abnormalities found in the noses submitted to revision rhinoplasty from the surgeon's point of view, but do not discuss the patient's aesthetic-functional complaints. During a preoperative evaluation, the lack of attention to the patient's complaint may induce unsatisfactory postoperative results and fall short of the patient's expectations.

Revision rhinoplasty is a procedure that requires greater experience and skill from the surgeon. Many of the cases are challenging in several aspects, not only aesthetic-functional, but also psychological ones, in the case of patients with high expectations, with complaints that are not in harmony with the findings on the preoperative examination, and even in patients with dysmorphic alterations who will rarely be satisfied with the postoperative results.^2^

No other aesthetic procedure requires such detailed preoperative evaluation as does the rhinoplasty. A thorough and well-documented examination associated with the knowledge of the patient's complaints and expectations is crucial.^2^

The study by Berger et al. demonstrates the importance of collecting ethnological, cultural and anthropometric data through a preoperative electronic protocol, aiming to evaluate the main indications for the surgical procedure, in addition to the main surgical maneuvers that should be used. Such organization allows the best surgical planning, aiming at the excellence of the surgical results, reducing the incidence of revision surgeries.^4^

Finally, as the basis for a successful physician-patient relationship, especially in a situation with greater demands and anxieties regarding the final outcome, the physician must be aware of the patients’ aesthetic and functional complaints, as well as their desires for change and expectations related to the procedure. The physician should recognize the objective deformities, correlating them with the patient's complaints aiming to propose to the patient the real possibilities of a new surgical intervention.^3^

## Objectives

The main objective of the study was to outline the main esthetic-functional complaints reported by patients to be submitted to a revision rhinoplasty in our hospital and to correlate them with the internal and external objective nasal evaluation performed by the surgeon. Other epidemiological and surgical questions were also investigated, such as the number of previous surgeries and the time since they were performed, previously used surgical accesses, and the reasons why the patient did not return to the previous surgeon.

## Methods

During the questionnaire development, it was decided to divide it into three parts: overall epidemiological questions, questions about the patients’ aesthetic and functional complaints and objective evaluation by the surgeons. The first part consisted of questions about the number of previous surgeries, the time elapsed since the last surgery, the surgeon who performed the last surgery, and the reasons why the patient sought another surgeon, if that occurred.

The second part consisted of questions to the patient (epidemiological issues, patient's functional and aesthetic concerns). The third part was exclusively used for the collection of information requested from the surgeon (aesthetic-functional objective evaluation of the nose).

In most questions asked to patients and physicians, there was the possibility of responding to more than one alternative, according to the alterations found – Questions 4, 6, 7 to 10, 12 to 16.

Aiming to better understand the patient regarding aesthetic complaints and for the adequate filling out of the questionnaire, the nose was divided into upper, middle, nasal tip and other regions.

The upper and middle regions were divided into high or low, broad or narrow, crooked (rhinoscoliosis), nasal bridge with an irregular appearance or other alterations mentioned by the interviewees concerning those regions.

The nose tip was subdivided into bulbous, narrow/pinched, upturned/raised (in the case of an excessive nasolabial angle), downturned (in the case of a nasolabial angle less than normal), prominent/protruding, asymmetrical, lacking appropriate definition, with collapse during inspiration and other changes mentioned by those interviewed about that region.

In the division regarding other regions the nasal base was evaluated – if broad or narrow; the columella – short or long; scar retractions – where all cases of inverted V were allocated, as well as cases of unsightly scar; visible graft and other alterations mentioned by the interviewees that could not be related to the upper, middle and nasal tip regions.

The study was prospectively carried out in a private Otorhinolaryngology Institution from June 2012 to November 2012, after being approved by the Ethics Committee of the institution under the Brazil platform number CEP CONEP CAAE 04901012.5.0000.5529 and registered under CEP 0012/2012.

After receiving information about the research and giving their authorization by signing the free and informed consent form, 43 patients with surgical indication of revision rhinoplasty were submitted to an interview prior to the surgery by the main investigator to fill out the questionnaire.

The third part of the questionnaire was then applied by the main investigator to the surgeon responsible for the surgery, preoperatively.

The patients included in the study had surgical programming of revision rhinoplasty, with mandatory aesthetic and optional functional purposes, having previously undergone rhinoplasty with the same or another surgeon.

The exclusion criteria included patients previously submitted to rhinoplasty that had undergone only functional corrections and those who did not agree to participate in the study.

The questionnaires were then tabulated and the patients’ aesthetic and functional concerns and the surgeons’ objective evaluations were compared.

According to the nature of the analyzed data, the statistical treatment considered to be adequate according to the nature of the analyzed data was performed. Using descriptive statistics, the frequencies related to the functional complaints and deformities observed by the surgeons were calculated. Using inferential statistics, the physicians’ and patients’ evaluations were analyzed for the different variables related to complaints and evaluations of nasal aesthetic, using the *χ*^2^ test. The significance level used for these variables was *p* < 0.05.

## Results

### Questions to the patient

#### Epidemiological questions

Among the assessed population, 29 patients were females (67.4%) and 14 were males (32.6%). The mean age was 30.6 years (range 16–51 years).

Of the 43 assessed patients, 88.4% (38) had been submitted to only one previous rhinoplasty. Only 3 patients (7%) had undergone 2 previous surgeries and 2 patients (4.6%) had been submitted to more than 3 previous rhinoplasties.

Most patients (62.7%) reported that the last surgery had been performed more than 12 months before. This period ranged from 13 months to 16 years, with a mean of 4.6 years. Only two patients had undergone surgery less than 6 months before the last procedure.

Most of the assessed patients (74.4%) had been previously submitted to surgery performed by the same surgeon. Only 11 patients (25.6%) had undergone a previous rhinoplasty by a different surgeon or surgeons (Table 1).

**Table 1:** Epidemiological questions.

Mean age, in years (range)	30.6 (16–51yrs)
Gender F/M (%)	67.4/32 (6)
*N*. of previous rhinoplasties, 1/2/3 (%)	88; 4/7.0/4 (6)
Time of last surgery <6 m, 6–12 m, >12 m (%)	4; 7/32.5/62 (8)
Surgeon who performed the last surgery, same/other (%)	74; 4/25 (6)

Among the 11 patients who had been operated by other surgeons, the main reported reason to seek another professional for the nasal correction was the fact that they did not consider the first result as satisfactory and did not feel safe with the previous surgeon (*n* = 6 or 54.5% of the patients). Among the 11 patients; 27.3% reported that they had had complications in the previous surgery and were afraid they would occur again if they were operated by the same surgeon.

**Figure 1:**
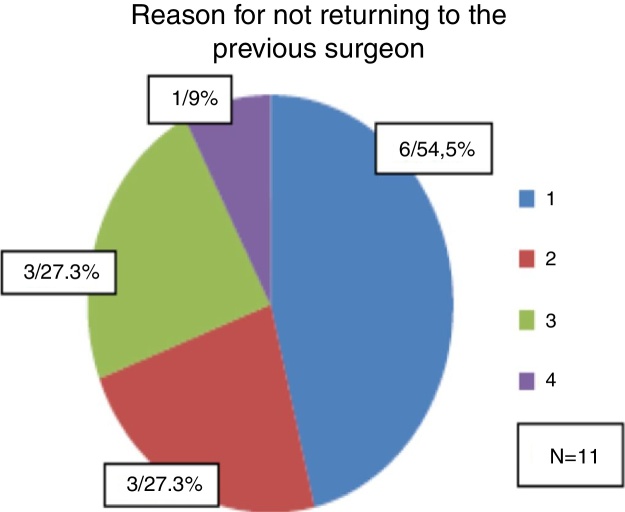
(1) I do not consider my first result a satisfactory one and I do not trust the previous surgeon; (2) I had complications in the previous surgery and I am afraid they will happen again if the surgery is performed by the same surgeon; (3) The previous surgeon no longer works for my health insurance company; (4) The value charged by the first surgeon for a new correction was excessive and the technique proposed by the chosen surgeon was more promising.

The same number of patients (27.3%) reported having sought another professional because the previous surgeon no longer worked for the patient's health insurance company. Only one patient (9%) reported that he sought another professional because the cost of the first surgeon for a new correction was very high and the technique proposed by the chosen surgeon was more promising (Fig. 1).

#### Functional concerns reported by the patient

Among the permanent alterations patients were asked about, 14 patients (32.5%) complained of one or more changes after the last surgery, corresponding to 26 complaints (Table 2).

**Table 2:** Permanently acquired alterations after rhinoplasty (*n* = 14).

Pain	Sensitivity	Swelling/edema	Bleeding	Nasal secretion	Crusts	Skin alteration	Others
3	5	4	4	2	4	3	1

The reported skin changes were changes to dry and oily skin and depigmentation in the bridge area. A complaint reported by one patient was permanent pain in the columella due to the reaction to a nylon suture.

Regarding respiratory complaints, 31 patients (72%) reported that their breathing was better or equal after the last surgery. Twelve patients complained of worsening of breathing since the last surgery, corresponding to 38% of the patients.

Also related to respiratory complaints, 16 patients (37.2%) complained of obstructive respiratory symptoms (Table 3).

**Table 3:** Obstructive respiratory symptoms (*n* = 16).

Nasal obstruction	Oral breathing	Nocturnal snoring	Dependence on nasal decongestants	Others (dry nose and coryza/nasal pruritus)
3	5	4	4	2

#### Aesthetic concerns reported by the patient

The most common subjective aesthetic complaint reported by the patients was the dropped nasal tip in 17 patients (39.5%), followed by the elevated bridge in the upper third in 14 or 32.5% of the patients and the broad nasal base in 9 patients or 20.9% (Table 4).

**Table 4:** Aesthetic complaints reported by the patient (*n* = 43).

Complaints – bridge	Upper third (NP)	Middle third (NP)	Complaints – nasal tip	NP	Complaints – other regions	NP
High	14	4	Bulbous	7	Broad nasal base	9
Low	1	1	Narrow	1	Narrowed nasal base	0
Wide	2	2	Upturned (excessive NLA)	1	Short columella	1
Narrow	0	0	Downturned (NLA below the normal)	17	Long columella	2
Crooked nose (rhinoscoliosis)	0	3	Projected	0	Scar retraction	2
Bridge irregularity	5	4	Asymmetric	5	Unsightly scar	1
Outros	0	0	Little definition	4	Visible/displaced graft	3
			Collapse on inspiration	2	Others	1 – Base asymmetry
			Others	0		

NLA, nasolabial angle; NP, number of patients.

### Questions to the surgeon

#### Nasal and intranasal assessment

The main access performed in the previous surgery was the closed access, which corresponds to 88.4% of the cases, 38 patients, with 3 (7%) patients having been submitted to the open technique in the previous surgery and in 2 the surgery was performed using delivery access (4,6%) (Table 5).

**Table 5:** Access performed in the previous surgery.

Closed	Open	Delivery
38	03	02

Regarding the objective intranasal assessment, surgeons reported abnornalities in anterior rhinoscopy examination in 20 patients. Of these alterations, the most frequent one was the presence of residual septal deviation observed in 11 patients of the 43 assessed (25.5%).

The other alterations observed were synechia, scar retraction, granuloma, turbinate hypertrophy, and collapse of the internal and external nasal valves (Table 6).

**Table 6:** Intranasal evaluation performed by the surgeon (*n* = 43).

NSF	Septal deviation	Turbinate hypertrophy	Synechia	INV collapse	Scar retraction	ENV collapse	Granuloma	Septal perforation
23	11 (25.6%)	6 (14%)	4 (9%)	3 (7%)	1 (2.3%)	1 (2.3%)	1 (2.3%)	0

NSF, no special features; INV, internal nasal valve; ENV, external nasal valve.

#### Objective evaluation of nasal aesthetics

The objective evaluation performed by the surgeons concerning the nasal aesthetics of the assessed patients showed as the main finding the drooping nasal tip in 19 patients (44.2%) followed by elevated nasal bridge in the upper third of the nose in 18 patients (41.8%) and irregularities on the bridge, bulbous nasal tip and widened nasal base, each accounting for 12 patients (27.9%) (Table 7).

**Table 7:** Objective nasal aesthetic evaluation performed by the surgeon (*n* = 43).

Bridge assessment	Upper third (NP)	Middle third (NP)	Tip assessment	NP	Assessment of other regions	NP
High	18	11	Bulbous	12	Broad nasal base	12
Low	2	2	Narrow	1	Narrowed nasal base	0
Wide	2	1	Upturned (excessive NLA)	0	Short columella	2
Narrow	0	0	Downturned (NLA below the normal)	19	Long columella	3
Crooked nose (rhinoscoliosis)	2	6	Projected	1	Scar retraction	9 inverted V (4)
Bridge irregularities	12	5	Asymmetric	3	Unsightly scar	0
Others	1 – open ceiling	0	Little definition	10	Visible/displaced graft	4
	1 – deep radix		Collapse on inspiration	3	Others	1 – long nose
	1 – incomplete bone fracture		Others	1 – Bifid tip		1 – Insufficient Nasal Spine
						1 – Crooked columella
						1 – Wide columella

NLA, nasolabial angle; NP, number of patients.

### Patient–physician assessment

#### Functional complaints

The intranasal evaluation performed by the physicians on the 16 patients who reported obstructive respiratory symptoms showed that residual septal deviation was the major cause of non-improvement in the assessed patients’ breathing, corresponding to 56.25% of these 9 patients.

The intranasal evaluation of patients who reported obstructive nasal symptoms are shown in Table 8.

**Table 8:** Intranasal evaluation of patients who reported obstructive nasal symptoms (*n* = 16).

NSF	Septal deviation	Turbinate hypertrophy	Synechia	INV collapse	Scar retraction	ENV collapse
2 (12.5%)	9 (56.25%)	4 (28.5%)	4 (28.5%)	3 (18.75%)	1 (6.25%)	1 (6.25%)

NSF, no special features; INV, internal nasal valve; ENV, external nasal valve.

#### Aesthetic complaints

When comparing the patients’ subjective complaints and the surgeons’ objective evaluations, drooping of the nasal tip and the presence of an elevated bridge in the upper third of the nose were the findings that were noted by both patients and physicians. These findings were concomitantly reported as a subjective complaint of patients and an objective assessment by the surgeon, respectively, in 34.9% (15) and 30.23% (13) of the assessed patients (Table 8). Nevertheless, it can be observed that the evaluated surgeons identified the drooping nasal tip in patients with a drooping nasal tip complaint in 78.9% of the cases. The presence of an elevated bridge in the upper third was identified by surgeons in 92.8% of patients with such subjective complaint.

Third, the presence of a broad nasal base was subjectively reported by the patients and was objectively verified by the surgeons in 6 patients (13.9%). In this case, surgeons agreed with the patients’ subjective assessment in 66.6% of the cases.

The bulbous tip had the fourth highest frequency of concordance among patients and surgeons and occurred in 5 patients (11.6%). In this case, the surgeons agreed with the patients’ subjective complaints in 71.4% of the cases (Table 9).

**Table 9:** Frequency of concomitant patient–physician findings (*n* = 43).

Bridge evaluation	Upper third (f)	*p*-value	Middle third (f)	*p*-value	Nasal tip evaluation	*f*	*p*-value	Evaluation of other regions	*f*	*p*-value
High	13	0.50	3	0.09	Bulbous	5	0.30	Broad nasal base	6	0.62
Low	1	1.0	1	1.0	Narrow	0	0.47	Narrowed nasal base	0	–
Wide	1	0.61	1	1.0	Upturned (excessive NLA)	0	1.0	Short columella	0	1.0
Narrow	0	1.0	0	1.0	Downturned (NLA below the normal)	15	0.83	Long columella	2	1.0
Crooked (rhinoscoliosis)	0	0.47	3	0.48	Projected	0	1.0	Scar retraction	2	0.05
Bridge irregularity	5	1.0	2	1.0	Asymmetric	3	0.71	Unsightly scar	0	1.0
Others	0	–	0	–	Little definition	4	0.14	Visible/displaced graft	3	1.0
					Collapse on inspiration	2	1.0	Others	0	–
					Others	0	–			

NLA, nasolabial angle; *f*, frequency.

## Discussion

Many of the alterations in the nasal anatomy found in patients undergoing revision rhinoplasty are difficult to manage, not only from a surgical point of view but also from a psychological one, when dealing with a patient previously dissatisfied with prior results and who, in most cases, has high expectations and anxieties that make it difficult for them to understand the intrinsic limitations of the procedure.

In most cases, the need for revision rhinoplasty is the result of a poorly performed prior evaluation, inappropriate patient selection, failure to adequately explain about the limitations related to surgery to the patient, and limitations in performing the surgical maneuvers during the procedure.^1^, ^5^

To optimize patient satisfaction after a revision surgery, the surgeon must be aware of the aesthetic and functional complaints reported by the patient, as well as perform a very detailed and objective nasal evaluation, to ensure that no alteration in nasal anatomy goes unnoticed and is not considered capable of being corrected during surgery.

The surgeon must validate the patient's aesthetic and functional complaints through a detailed external and internal evaluation of the nose. The physician should make every effort to “see what the patient sees in himself;” otherwise, a doctor-patient trust relationship will be less than optimal.^3^

Most of the patients evaluated in this study (62.8%) underwent a new rhinoplasty 12 months after the last surgery. This time ranged from 13 months to 16 years, with a mean of 4.6 years.

Although widely disseminated among surgeons, the knowledge that soft tissue takes approximately one year to return to the preoperative state is not always true.^2^

This is demonstrated by the fact that some patients spend years satisfied with their postoperative results and after several years complain again of visible aesthetic deformities. This fact results from the scar retraction, which is gradual and continues for years after the first surgical procedure.^2^

Among the most common aesthetic complaints made by the patients in this study, a drooping nasal tip, presence of residual nasal hump and broad nasal base were the three most prevalent complaints among the 43 assessed patients (Table 4).

The objective evaluation of the nasal aesthetic performed by the surgeon showed a greater number of visualized alterations when compared with the patients’ complaints. Nevertheless, the drooping of the nasal tip and the presence of residual nasal hump were the two most prevalent factors in the surgeons’ evaluation reports.

Other alterations reported by surgeons, but less frequently than the previous ones, were the presence of a broad nasal base, a bulbous tip and bridge irregularities in the upper third of the nose with the same frequency of reports, followed by the raised bridge in the middle third, nasal tips with little definition and scar retractions (Table 7).

In most evaluations, the objective reports of surgeons showed a higher frequency of findings than the nasal aesthetic complaints made by the patients. The evaluated patients noticed only 48.6% of the aesthetic deformities reported by the surgeons.

This fact is fully understood, given the medical knowledge of the correct nasal anatomy, the professionals’ experience in the systematic nasal evaluation when the visual and tactile stimuli are associated during the physical examination, in addition to the need for the detailed evaluation of all the aesthetic deformities of a patient previously submitted to a rhinoplasty.

This difference between the assessments made by patients and surgeons is consistent with the findings of Tobin and Webster et al., that showed patients are less critical regarding their postoperative appearance when compared to the surgeons responsible for their surgeries.^6^

When crossing the data from the aesthetic evaluation performed by the physicians and the aesthetic complaints of the assessed patients, the presence of a drooping nasal tip and residual nasal hump were the two main complaints of patients confirmed by the surgeons, followed by the broad nasal base, bulbous tip and irregularities in the upper third of the nose – with the same frequency – and nasal tips with little definition.

Such findings contrast with the results of other studies such as Pearlman et al., where the main complaint of previously operated patients reported in the objective examination by the physicians is the presence of an asymmetric tip.^7^

This fact may be due to the lower frequency of nasal tip intervention in surgeries performed in our country, where the closed technique was predominant and used in 88.4% of primary rhinoplasties.

Although the concomitant findings of patients and surgeons suggest a greater concern of both regarding more easily visible aesthetic deformities such as the nasal hump and the nasal tip, it was not possible to identify a statistically significant correlation (*p* < 0.05), as shown in Table 9.

Regarding the subjective functional complaints reported by the patients, 37.2% of them complained of some type of nasal obstructive symptoms: nasal obstruction, mouth breathing, nocturnal snoring, dependence on nasal decongestants or others. The frequency found in our study is below that found in studies available in the international literature as in the study by Thomson and Mendelson et al.,^8^ where 59% to 68% of patients reported nasal obstructive symptoms after revision rhinoplasty.

These data suggest the more attention paid by the surgeons in our country to nasal respiratory complaints in the preoperative period, greater dedication during the surgical procedure to prevent subsequent functional deformities and the use of more controlled techniques to prevent creating functional complications in patients submitted to primary rhinoplasty.

Among the 16 patients with functional respiratory alteration complaints, only two (12.5%) were not identified by their surgeons as having intranasal deformities that would justify their complaints (Table 8).

A correlation between subjective obstructive symptoms and the intranasal assessment performed by surgeons was present in 87.5% of cases with one or more nasal obstructive symptoms. Among the patients with respiratory symptoms, the main deformity found was residual septal deviation (56.25%), followed by turbinate hypertrophy and synechiae, both observed in 28.5% of the patients, and the collapse of the internal nasal valve in 19.75% of patients with obstructive nasal complaints.

In the international literature, nasal valve collapse is reported as the second most common cause of postoperative nasal obstruction, second only to the presence of residual septal deviation as demonstrated by Pearlman et al.^7^

In this study, most revision rhinoplasties were performed by the same surgeon who had performed the primary surgery (74.4% of the cases). Among the 25.6% of patients undergoing revision surgery performed by a new surgeon, the main reasons for seeking another professional were the fact that the patient did not consider the result of the first procedure satisfactory and did not feel safe with the previous surgeon or had had complications in the previous surgery and was afraid they would happen again if the surgery was performed by the same surgeon.

This fact suggests that the search for a surgeon with greater skill and knowledge of improved techniques is the main reason why patients seek other professionals when they need a surgical revision.

## Conclusion

The downturned nasal tip followed by residual bridge hump was the main complaints reported by the patients and confirmed by the objective examination by the physicians. Such findings differ from those found in other studies, where the asymmetric tip and bridge irregularities in the middle third are the most frequent complaints and findings observed by surgeons. The presence of 37.2% of the patients with nasal obstructive complaints shows that greater attention should be paid to functional deformities during the first surgical procedure to prevent reinterventions aimed to correct these deformities. The differences observed between patients’ complaints and surgeons’ evaluations confirm the need for detailed assessment and clarification to the patients regarding their expectations and actual surgical possibilities.

Some important research factors suggest further investigation. For future research, the division between groups previously operated by the same surgeon and groups operated by other surgeons could disclose new data, as well as an increase in the number of assessed patients aiming to validate the study in a statistically significant manner.

## Conflicts of interest

The authors declare no conflicts of interest.
